# Supplementary material for: An Integrative lifecycle design approach based on carbon intensity for renewable-battery-consumer energy systems
Source: Commun Eng. 2025 Jan 30;4:14. doi: 10.1038/s44172-024-00339-5 (PMC11782646; doi:10.1038/s44172-024-00339-5)
Supplement: Supplementary file 2 — Supplementary information [file 44172_2024_339_MOESM2_ESM.pdf]

Supplementary information for

An Integrative lifecycle design approach for renewable-battery-consumer energy systems

Song Aoye [1,2], Zhou Yuekuan [1,2,3,4*]

[1]*Sustainable Energy and Environment Thrust, Function Hub, The Hong Kong University of Science and Technology (Guangzhou), Nansha, Guangzhou, 511400, Guangdong, China*

[2]*Division of Emerging Interdisciplinary Areas, The Hong Kong University of Science and Technology, Clear Water Bay, Hong Kong SAR, China*

[3]*Department of Mechanical and Aerospace Engineering, The Hong Kong University of Science and Technology, Clear Water Bay, Hong Kong SAR, China*

[4]*HKUST Shenzhen-Hong Kong Collaborative Innovation Research Institute, Futian, 518048, Shenzhen, China*

*Corresponding author:

Email: yuekuanzhou@hkust-gz.edu.cn (Zhou Y.)

This file includes:

Supplementary Tables 1 to 14,

Supplementary Figures 1 to 3,

Supplementary Note 1.

Supplementary Table 1 The electrical performance of the installed PV module[1].

Electrical performance parameters (STC[a])			
Type	Hiku-CS3W-450 MS	Operation temperature	-40~85 °C
Maximum power (P_{max})	450 W	Size	2,108×1,048×35 mm
Voltage at maximum power (V_{mp})	41.1 V	Temperature coefficient (Pmax)	-0.34% / °C
Current at maximum power (I_{mp})	10.96 A	Temperature coefficient (Pmax)	-0.26% / °C
Open-circuit voltage (V_{oc})	49.1	Temperature coefficient (Pmax)	0.05% / °C
Short-circuit current (I_{sc})	11.60 A	Normal Operating Cell Temperature	41±3 °C
Efficiency	20.39%		

[a] Standard testing conditions (STC): Solar radiation at 1,000 W m^{-2}, Cell temperature at 25 °C and AM 1.5 Standard Spectrum.

Supplementary Table 2 Polynomial coefficients for battery degradation curves at different depth of discharges (DoDs)[2].

	DoD = 1	*DoD* = 0.8	*DoD* = 0.6	*DoD* = 0.4	*DoD* = 0.3	*DoD* = 0.2	*DoD* = 0.1
k_1	-2.685×10^{-11}	-8.732×10^{-12}	-2.562×10^{-12}	-5.362×10^{-13}	-3.084×10^{-13}	-1.934×10^{-13}	-1.292×10^{-13}
k_2	1.539×10^{-7}	6.271×10^{-8}	2.665×10^{-8}	9.537×10^{-9}	6.622×10^{-9}	4.866×10^{-9}	3.727×10^{-9}
k_3	-3.261×10^{-4}	-1.947×10^{-4}	-1.276×10^{-4}	-7.764×10^{-5}	-6.492×10^{-5}	-5.581×10^{-5}	-4.894×10^{-5}
k_4	1	1	1	1	1	1	1

Supplementary Table 3 Electricity price for residential buildings in Guangzhou.

$C_{eg,imp1}$[a] (CN¥ kWh^{-1})[b] (from May to October)				$C_{eg,imp2}$[c] (CN¥ kWh^{-1}) (from November to April)			
E_{imp} (each month per household, kWh)	Peak[d]	Flat[e]	Valley[f]	E_{imp} (each month per household, kWh)	Peak[d]	Flat[e]	Valley[f]
≤ 260	0.9950	0.5889	0.2292	≤ 200	0.9950	0.5889	0.2292
> 260 and ≤ 600	1.0450	0.6389	0.2792	> 200 and ≤ 400	1.0450	0.6389	0.2792
> 600	1.2950	0.8889	0.5292	> 400	1.2950	0.8889	0.5292

[a] $C_{eg,imp1}$ is the import cost from May to October (summer period).

[b] 1 CN¥=0.1429 US$

[c] $C_{eg,imp2}$ is the import cost from November to April (non-summer period).

[d] Peak period is from 10:00 AM to 12:00 PM and from 14:00 PM to 19:00 PM.

[e] Flat period is from 8:00 AM to 10:00 AM, from 12:00 PM to 14:00 PM and from 19:00 PM to 24:00 PM.

[f] Valley period is from 0:00 AM to 8:00 AM.

Supplementary Table 4 Electricity price for commercial users in Guangzhou.

Period	Super peak[a]	Peak[b]	Flat[c]	Valley[d]
$C_{eg,imp,c}$ (CN¥ kWh^{-1})[e]	1.7156	1.3780	0.8220	0.3296

[a] Super peak period is from 11:00 AM to 12:00 PM and from 15:00 PM to 17:00 PM in July, August, and September.

[b] Peak period is from 10:00 AM to 11:00 AM, from 14:00 PM to 15:00 PM and from 17:00 PM to 19:00 PM in July, August, and September. In other periods (except for July, August, and September), the peak period is from 10:00 AM to 12:00 AM and from 14:00 PM to 19:00 PM.

[c] Flat period is from 8:00 AM to 10:00 AM, from 12:00 PM to 14:00 PM and from 19:00 PM to 24:00 PM.

[d] Valley period is from 0:00 AM to 8:00 AM.

[e] 1 CN¥=0.1429 US$

Supplementary Table 5 Electricity price for residential buildings in Shenzhen.

$C_{eg,imp1}$[a] (CN¥kWh^{-1})[b] (from May to October)				$C_{eg,imp2}$[c] (CN¥ kWh^{-1}) (from November to April)			
E_{imp} (one month per household, kWh)	Peak[d]	Flat[e]	Valley[f]	E_{imp} (one month per household, kWh)	Peak[d]	Flat[e]	Valley[f]
≤ 260	1.1208	0.6629	0.2573	≤ 200	1.1208	0.6629	0.2573
> 260 and ≤ 600	1.1708	0.7129	0.3073	> 200 and ≤ 400	1.1708	0.7129	0.3073
> 600	1.4208	0.9629	0.5573	> 400	1.4208	0.9629	0.5573

[a] $C_{eg,imp1}$ is the import cost from May to October (summer period).

[b] 1 CN¥=0.1429 US$

[c] $C_{eg,imp2}$ is the import cost from November to April (non-summer period).

[d] Peak period is from 10:00 AM to 12:00 PM and from 14:00 PM to 19:00 PM.

[e] Flat period is from 8:00 AM to 10:00 AM, from 12:00 PM to 14:00 PM and from 19:00 PM to 24:00 PM.

[f] Valley period is from 0:00 AM to 8:00 AM.

Supplementary Table 6 Electricity price for commercial users in Shenzhen.

Period	Super peak[a]	Peak[b]	Flat[c]	Valley[d]
$C_{eg,imp,c}$ (CN¥ kWh^{-1})[e]	1.7156	1.3780	0.8220	0.3296

[a] Super peak period is from 11:00 AM to 12:00 PM and from 15:00 PM to 17:00 PM in July, August, and September.

[b] Peak period is from 10:00 AM to 12:00 AM and from 14:00 PM to 19:00 PM except for the super peak period.

[c] Flat period is from 8:00 AM to 10:00 AM, from 12:00 PM to 14:00 PM and from 19:00 PM to 24:00 PM except for super peak period.

[d] Valley period is from 0:00 AM to 8:00 AM.

[e] 1 CN¥=0.1429 US$

Supplementary Table 7 Electricity price for residential buildings in Shanghai.

$C_{eg,imp}$ (CN¥ kWh^{-1})[a]		
E_{imp} (kWh per·household)	Peak[b]	Valley[c]
≤ 3,120	0.617	0.307
> 3,120 and ≤ 4,800	0.677	0.337
> 4,800	0.977	0.487

[a] 1 CN¥=0.1429 US$

[b] Peak period is from 6:00 AM to 22:00 PM.

[c] Valley period is from 22:00 PM to 6:00 AM (next day).

Supplementary Table 8 Electricity price for commercial users in Shanghai.

Period	Peak[a]	Valley[b]
$C_{eg,imp,c}$ (CN¥kWh^{-1})[c]	0.9753	0.4739

[a] Peak period is from 6:00 AM to 22:00 PM.

[b] Valley period is from 22:00 PM to 6:00 AM (next day).

[c] 1 CN¥=0.1429 US$

Supplementary Table 9 Electricity price for residential buildings in Kunming.

E_{imp} (kWh per·household)	$C_{eg,imp}$ (CN¥ kWh^{-1})[a]
≤ 1,560	0.3336
> 1,560 and ≤ 3,600	0.4236
> 3,600 and ≤ 4,680	0.4736
> 4,680	0.7736

[a] 1 CN¥=0.1429 US$

Supplementary Table 10 Electricity price for commercial users in Kunming.

Period	Peak[a]	Flat[b]	Valley[c]
$C_{eg,imp}$ (CN¥ kWh^{-1})[d]	0.6998	0.4878	0.2758

[a] Peak period is from 9:00 AM to 12:00 PM and from 18:00 PM to 23:00 PM.

[b] Flat period is from 7:00 AM to 9:00 AM, from 12:00 PM to 18:00 PM.

[c] Valley period is from 23:00 PM to 0:00 AM and from 0:00 AM to 7:00 AM.

[d] 1 CN¥=0.1429 US$

Supplementary Table 11 Electricity price for residential buildings in Beijing.

E_{imp} (each month per household, kWh)	≤ 240	> 240 and ≤ 400	> 400
$C_{eg,imp,r}$ (CN¥ kWh^{-1})[a]	0.4883	0.5383	0.7883

[a] 1 CN¥=0.1429 US$

Supplementary Table 12 Electricity price for commercial users in Beijing.

Period	Super peak[a]	Peak[b]	Flat[c]	Valley[d]
$C_{eg,imp,c}$ (CN¥ kWh^{-1})[e]	1.2713	1.1574	0.8617	0.5953

[a] Super peak period is from 11:00 AM to 13:00 PM and from 16:00 PM to 17:00 PM in July and August.

[b] Peak period is from 10:00 AM to 11:00 AM, from 13:00 PM to 15:00 PM and from 18:00 PM to 21:00 PM in July and August. In other periods (except for July and August), the peak period is from 10:00 AM to 15:00 PM and from 18:00 PM to 21:00 PM.

[c] Flat period is from 7:00 AM to 10:00 AM, from 15:00 PM to 18:00 PM and from 21:00 PM to 23:00 PM.

[d] Valley period is from 23:00 PM to 7:00 AM.

[e] 1 CN¥=0.1429 US$

Supplementary Table 13 Electricity price for residential buildings in Shenyang.

E_{imp} (kWh per·household annually)	$C_{eg,imp}$ (CN¥ kWh^{-1})[a]
≤ 2,640	0.5
> 2,640 and ≤ 3,720	0.55
> 3,720	0.8

[a] 1 CN¥=0.1429 US$

Supplementary Table 14 Electricity price for commercial users in Shenyang.

Period	Super peak[a]	Peak[b]	Flat[c]	Valley[d]
$C_{eg,imp,c}$ (CN¥ kWh^{-1})[e]	1.314	1.0584	0.7169	0.3754

[a] Super peak period is from 17:00 PM to 19:00 PM.

[b] Peak period is from 7:30 AM to 11:30 AM and from 19:00 PM to 21:00 PM.

[c] Flat period is from 5:00 AM to 7:30 AM, from 11:30 AM to 17:00 PM and from 21:00 PM to 22:00 PM.

[d] Valley period is from 22:00 PM to 5:00 AM.

[e] 1 CN¥=0.1429 US$

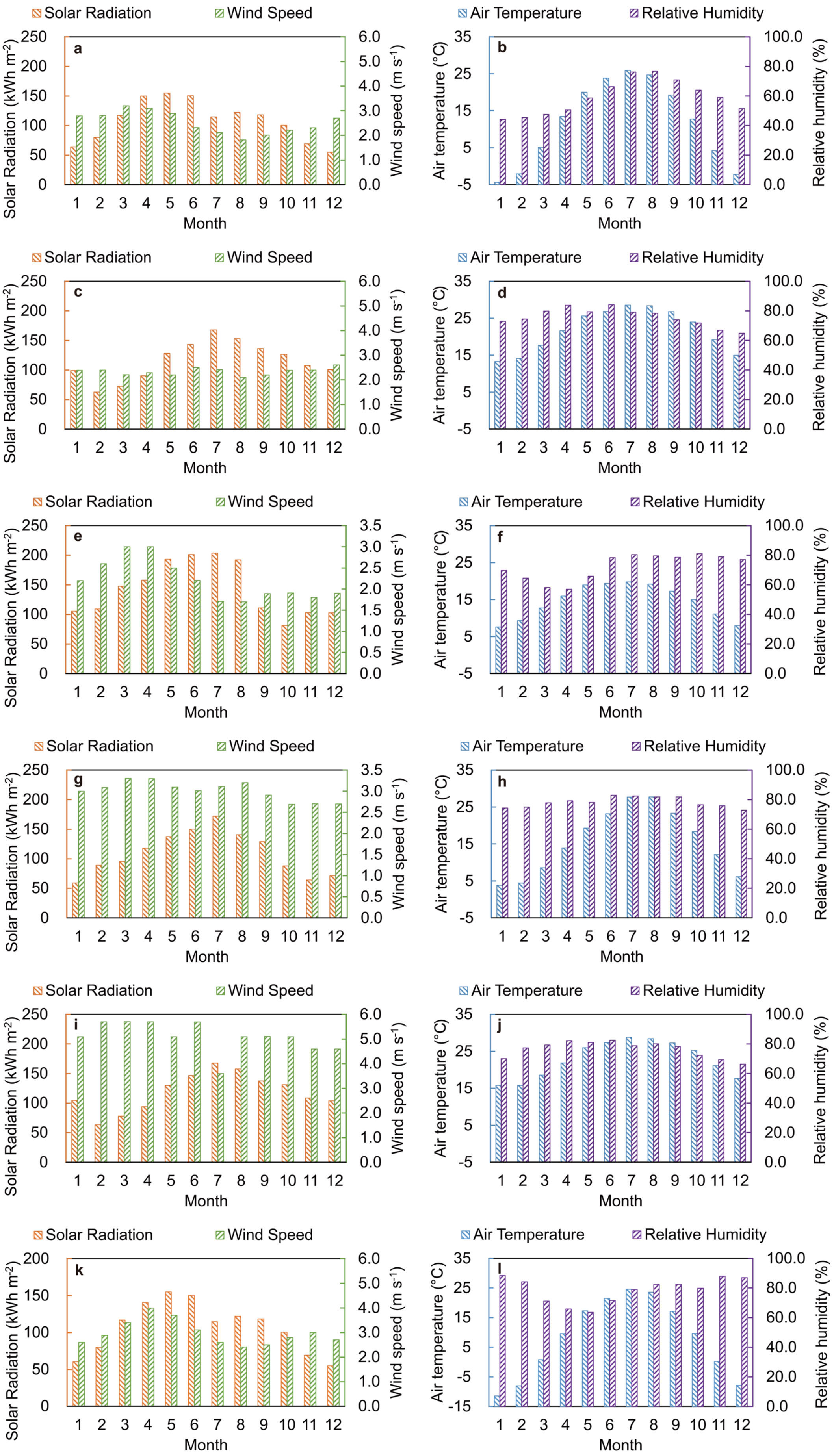
Solar Radiation
Wind Speed
Air Temperature
Relative Humidity
a
b
c
d
e
f
g
h
i
j
k
l
Solar Radiation (kWh m-2)
Wind speed (m s-1)
Air temperature (°C)
Relative humidity (%)
Month

Supplementary Figure 1 The climate information of different regions[3]. The monthly solar radiation and monthly average wind speed in Beijing (a), Guangzhou (c), Kunming (e), Shanghai (g), Shenzhen (i), and Shenyang (k); The monthly average air temperature and relative humidity in Beijing (b), Guangzhou (d), Kunming (f), Shanghai (h), Shenzhen (j), and Shenyang (l).

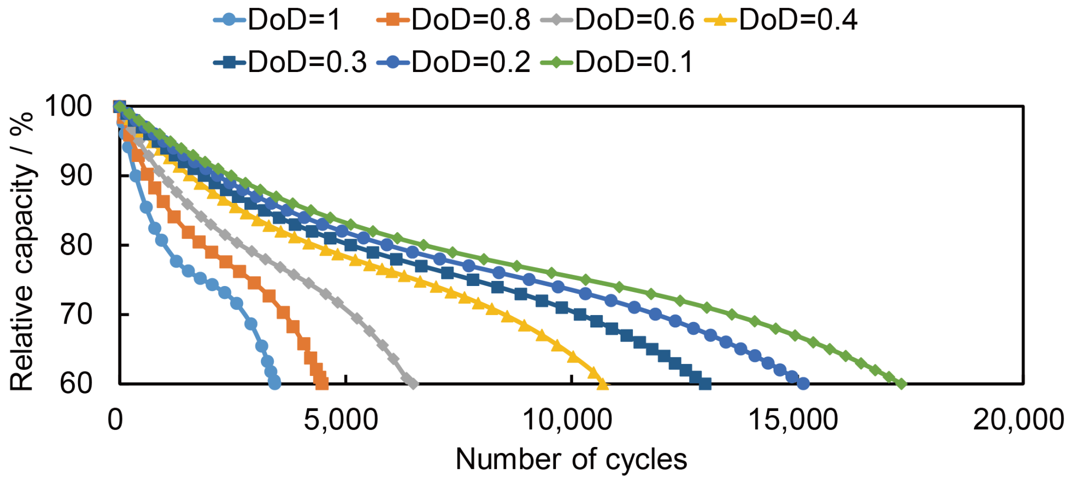

Supplementary Figure 2 Evolution of Li-ion battery relative capacity with different depths of discharge (DoDs) and number of cycles[2].

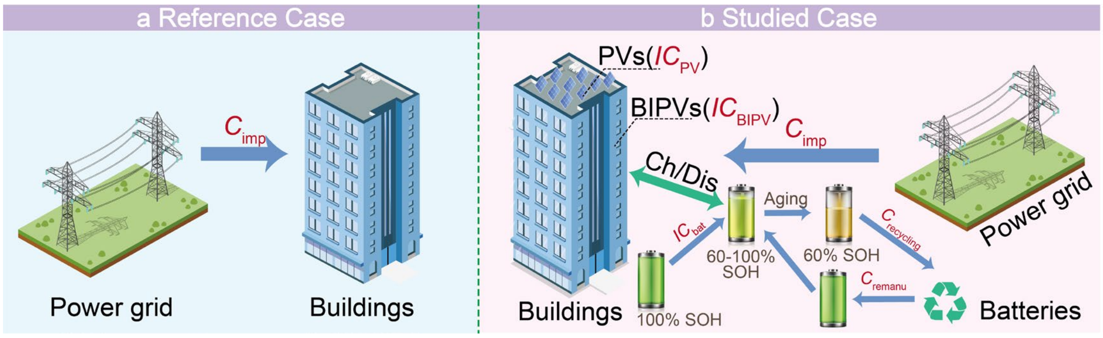

Supplementary Figure 3 The net present value calculation method for (a) the reference case and (b) studied cases. (Note: The C_{imp} refers to the cost from importing grid electricity, C_{recyc} refers to the recycling cost of End-of-Life batteries, C_{remanu} refers to the cost of remanufacturing new batteries from recycled batteries, IC_{PV} is the initial investment of BIPVs (building-integrated photovoltaics) and Solar PVs (photovoltaics), IC_{bat} refers to the initial investment cost of batteries, SOH refers to the state of health of batteries.)

Supplementary Note 1 System cost sources for the reference case and studied cases.

The following Equations (1)-(8) show supplementary Equations for NPV calculations[4]:

$$\Delta C_{\text{imp,save}}=\sum_{n=1}^{20}\frac{\int_0^{8760}\left[P_{\text{imp,Ref Case},n}(t)\cdot C_{\text{imp}}(t)\right]dt-\int_0^{8760}\left[P_{\text{imp,Study Case},n}(t)\cdot C_{\text{imp}}(t)\right]dt}{(1+r)^n}(1+\eta)^n \quad (1)$$

$$\Delta C_{\text{recyc}}=C_{\text{recyc,Study Case}}-C_{\text{recyc,Ref Case}}=\sum_{n=1}^{20}\frac{\sum_{i=1}^{j}C_{\text{recyc,Study Case,i},n}-\sum_{i=1}^{j}C_{\text{recyc,Ref Case},i,n}}{(1+r)^n} \quad (2)$$

$$\Delta C_{\text{remanu}}=C_{\text{remanu,Study Case}}-C_{\text{remanu,Ref Case}}=\sum_{n=1}^{20}\frac{\sum_{i=1}^{j}C_{\text{remanu,Study Case},i,n}-\sum_{i=1}^{j}C_{\text{remanu,Ref Case},i,n}}{(1+r)^n} \quad (3)$$

$$\Delta C_{\text{O\&M}}=C_{\text{O\&M,Study Case}}-C_{\text{O\&M,Ref Case}}=$$
$$\sum_{n=1}^{20}\frac{\sum_{i=1}^{j}C_{\text{O\&M-battery,Study Case},i,n}+C_{\text{O\&M-RE,Study Case},n}-\sum_{i=1}^{j}C_{\text{O\&M-battery,Ref Case},i,n}-C_{\text{O\&M-RE,Ref Case},n}}{(1+r)^n} \quad (4)$$

$$\Delta IC_{\text{PV}}=Cap_{\text{PV, Study Case}}\cdot C_{\text{PV}} \quad (5)$$

$$\Delta IC_{\text{bat}}=Cap_{\text{bat, Study Case}}\cdot C_{\text{bat}} \quad (6)$$

The subscript n refers to the nth year. i refers to the ith battery and j represents the last battery. The variables η and r is the escalation rate of grid electricity and the interest rate with a value of 1.4% and 2% [5], respectively. $P_{\text{imp}}(t)$ refers to the grid import power. C_{imp} (t) refers to the electricity price of the electricity grid, which is shown in Supplementary Tables 3-14. C_{recyc} (57 \$ kWh^{-1}) [6] and C_{remanu} (40 \$ kWh^{-1}) [7] refer to the recycling and remanufacturing cost for building static batteries. $C_{\text{O\&M}}$ refers to the operation and maintenance cost, which is 0.5% for batteries and 5% for renewable systems annually [8, 9, 10, 11, 12]. $Cap_{\text{PV,Study Case}}$ is the total installed capacity of BIPVs and PVs in studied cases. C_{PV} refers to the price of BIPVs and PVs (240 \$ kWp^{-1}). $Cap_{\text{bat, Study Case}}$ is the installed capacity of static batteries in studied cases. C_{bat} refers to the price of batteries (139 \$ kWh^{-1}) [13].

Figure S2 shows the cost sources included in the Reference Case and studied cases. The cost in the Reference Case is only the import cost (C_{imp}) of grid power, while the cost of the studied case includes the initial cost of PVs and BIPVs (IC_{PV} and IC_{BIPV}), the import cost of grid power (C_{imp}), the initial investment cost (IC_{bat}), recycling cost ($C_{\text{recycling}}$) and remanufacturing cost (C_{remanu}) of the battery.

Supplementary References

1. Csisolar. Hiku-CS3W-450MS, https://static.csisolar.com/wp-content/uploads/sites/9/2019/12/07115154/CS-Datasheet-HiKu_CS3W-MS_v5.9_CN.pdf. (2019).

2. Song Aoye, Zhou Yuekuan. A hierarchical control with thermal and electrical synergies on battery cycling ageing and energy flexibility in a multi-energy sharing network. *Renewable Energy* **212**, 1020-1037 (2023).

3. Meteotest. Meteonorm 8, Asia, https://meteonorm.com/. (2003).

4. Song Aoye, Zhou Yuekuan. Advanced cycling ageing-driven circular economy with E-mobility-based energy sharing and lithium battery cascade utilisation in a district community. *Journal of Cleaner Production* **415**, (2023).

5. Zhou Yuekuan, Cao Sunliang, Hensen Jan L. M. An energy paradigm transition framework from negative towards positive district energy sharing networks—Battery cycling aging, advanced battery management strategies, flexible vehicles-to-buildings interactions, uncertainty and sensitivity analysis. *Applied Energy* **288**, (2021).

6. Lander L., *et al.* Financial viability of electric vehicle lithium-ion battery recycling. *iScience* **24**, 102787 (2021).

7. Murdock Beth E., Toghill Kathryn E., Tapia‐Ruiz Nuria. A Perspective on the Sustainability of Cathode Materials used in Lithium‐Ion Batteries. *Advanced Energy Materials* **11**, (2021).

8. Dhundhara Sandeep, Verma Yajvender Pal, Williams Arthur. Techno-economic analysis of the lithium-ion and lead-acid battery in microgrid systems. *Energy Conversion and Management* **177**, 122-142 (2018).

9. Sarkar Tathagata, Bhattacharjee Ankur, Samanta Hiranmay, Bhattacharya Konika, Saha Hiranmay. Optimal design and implementation of solar PV-wind-biogas-VRFB storage integrated smart hybrid microgrid for ensuring zero loss of power supply probability. *Energy Conversion and Management* **191**, 102-118 (2019).

10. Mehrjerdi Hasan. Modeling, integration, and optimal selection of the turbine technology in the hybrid wind-photovoltaic renewable energy system design. *Energy Conversion and Management* **205**, (2020).

11. Veilleux Gabriel*, et al.* Techno-economic analysis of microgrid projects for rural electrification: A systematic approach to the redesign of Koh Jik off-grid case study. *Energy for Sustainable Development* **54**, 1-13 (2020).

12. Berrueta Alberto, Heck Michael, Jantsch Martin, Ursúa Alfredo, Sanchis Pablo. Combined dynamic programming and region-elimination technique algorithm for optimal sizing and management of lithium-ion batteries for photovoltaic plants. *Applied Energy* **228**, 1-11 (2018).

13. BloombergNEF. Battery Prices Are Falling Again as Raw Material Costs Drop. (2023).
